# District-level joint risk assessment of highly pathogenic avian influenza H5N1 at the human–animal–environment interface in live bird markets of Bogor, Indonesia

**DOI:** 10.14202/vetworld.2026.210-223

**Published:** 2026-01-20

**Authors:** Etih Sudarnika, Herwin Pisestyani, Syafrison Idris, Gunawan Setiaji, Dinda Iryawati, Nurul Hardianti, Ni Luh Putu Ika Mayasari, Okti Nadia Poetri, Chaerul Basri, Yusuf Ridwan, Srihadi Agungpriyono

**Affiliations:** 1 School of Veterinary Medicine and Biomedical Sciences, IPB University, Bogor 16680, Indonesia; 2 Global Health Agromaritime-One Health Collaborating Center, IPB University, Bogor 16680, Indonesia; 3 Directorate General of Livestock and Animal Health Services, Ministry of Agriculture of the Republic of Indonesia, Jakarta 12550, Indonesia; 4 Faculty of Medicine, IPB University, Bogor 16680, Indonesia

**Keywords:** avian influenza, joint risk assessment, live bird markets, One Health, poultry trade networks, public health risk, subnational surveillance, zoonotic disease

## Abstract

**Background and Aim:**

Highly pathogenic avian influenza (HPAI) A(H5N1) continues to be endemic in Indonesia, with live bird markets (LBMs) serving as key points for zoonotic transmission. While national assessments exist, there is a lack of local joint risk assessments (JRAs) specifically focused on LBMs. This study conducted the first district-level, multisectoral JRA of HPAI H5N1 at the human–animal–environment interface in LBMs of Bogor District and Municipality, Indonesia, utilizing the FAO–WHO–WOAH JRA Operational Tool adapted for subnational use.

**Materials and Methods:**

A qualitative, participatory JRA was carried out through a structured five-stage process, including governance formation, risk pathway development, stakeholder validation, technical risk analysis, and final consultation. In total, fifty stakeholders from sectors such as human health, animal health, environmental, trade, market, academic, and local government took part. Evidence was triangulated from poultry movement logs, animal and human surveillance data (Integrated Animal Health Information System [iSIKHNAS] and Early Warning and Response System [Sistem Kewaspadaan Dini dan Respons; SKDR), environmental sampling at 15 LBMs, trader interviews, and prior market studies. The risk analysis concentrated on the likelihood, impact, and uncertainty of human H5N1 infection over a 12-month period.

**Results:**

More than 90% of approximately 25 million poultry supplied annually to LBMs originated locally, with marked seasonal surges during religious festivals. Risk pathways highlighted poultry trade networks, market handling practices, slaughtering activities, and inadequate sanitation as key transmission nodes. Two environmental samples tested positive for influenza A, but no H5 subtype or human cases were detected locally since 2017. Consensus-based risk estimation classified the likelihood of at least one human H5N1 infection as low, with minor potential population-level impact. Uncertainty was rated moderate due to limited wild bird surveillance, incomplete environmental sampling, and variable data quality across sectors.

**Conclusion:**

This district-level JRA identified LBMs as persistent but manageable risk nodes for HPAI H5N1 transmission in Bogor. While the current risk to human health was assessed as low, structural and behavioral vulnerabilities justify proactive mitigation. Priority actions include strengthening LBM biosecurity, improving waste management, enhancing environmental surveillance, and reinforcing integrated One Health coordination. The study demonstrates the feasibility and policy relevance of locally implemented JRAs and provides an operational model for decentralized zoonotic risk assessment in endemic settings.

## INTRODUCTION

Avian influenza (AI), particularly highly pathogenic avian influenza (HPAI) A(H5N1), remains a major global zoonotic threat, affecting domestic and wild birds, a wide range of mammalian species, and occasionally humans. Global surveillance by the Food and Agriculture Organization (FAO), the World Health Organization (WHO), and the World Organization for Animal Health (WOAH) has documented a sustained increase in outbreaks across multiple host species. Between October 2023 and January 2025, a total of 4,206 HPAI A(H5N1) events were reported worldwide, including 3,195 in birds, 964 in mammals, and 43 in humans [1]. Although a joint FAO–WHO–WOAH risk assessment published in December 2024 classified the overall global public health risk as low, regions with high poultry density, active live bird markets (LBMs), and complex poultry trading networks remain at elevated risk, depending on the effectiveness of implemented mitigation measures [2].

Indonesia is a high-risk setting where AI remains endemic and continues to pose substantial public health and economic challenges. In 2023, the HPAI A(H5N1) clade 2.3.4.4b was detected for the first time in unvaccinated waterfowl, raising significant epidemiological concern because of its rapid dissemination and documented spillover into mammalian hosts, including viral isolates carrying mutations associated with mammalian adaptation [3, 4]. In Indonesia, HPAI is classified as a strategic animal disease [3, 5] and a national zoonotic priority [6], underscoring the need for strengthened surveillance systems and sustained control efforts.

At the subnational level, Bogor District is among Indonesia’s largest poultry-producing regions. In 2023, the recorded poultry population in Bogor included 27,837,593 broiler chickens, 9,496,122 layer chickens, 1,874,872 native or local chickens, 117,193 ducks, and 168,805 Muscovy ducks [7]. Active LBMs, long recognized as critical nodes for zoonotic amplification and human exposure, operate in both the Bogor District and the Bogor Municipality [8–10]. Although the most recent human H5N1 case in Bogor was reported in 2017, the sustained circulation of poultry and ongoing market activities underscore the ongoing public health relevance. Bogor was selected not only for its high poultry density but also for its central role in regional poultry trading networks, making it a strategic location for localized HPAI risk assessment.

To address these risks, the Indonesian government has established a multisectoral framework for joint risk assessment (JRA) of zoonotic diseases, as outlined in the Coordinating Ministry for Human Development and Culture Regulation No. 7/2022 on Guidelines for the Prevention and Control of Zoonoses and Emerging Infectious Diseases [11]. This framework aligns with the FAO–WOAH–WHO approach to zoonotic disease risk assessment [12] and emphasizes integrating human, animal, and environmental health sectors within a One Health framework for risk assessment, management, and communication. In addition, Circular Letter No. PM.03.01/C/28/2025, issued by the Directorate General of Disease Prevention and Control, reinforces the importance of strengthened coordination between the human and animal health sectors and highlights the need for early detection of AI cases in humans [13].

Despite Indonesia’s long-standing endemic presence of HPAI A(H5N1) and the known importance of LBMs as key sites for zoonotic transmission, comprehensive local-level risk assessments are limited. Most existing evaluations are conducted at national or regional levels and use aggregated surveillance data, which can hide variations in poultry movement, market practices, and human–animal–environment interactions within smaller areas. Specifically, JRAs at the district or market-level that apply the FAO–WHO–WOAH framework in real-world settings is rare, and few studies combine multisectoral evidence from animal health, public health, environmental monitoring, and market operations. Additionally, implementing a One Health approach locally is often hindered by fragmented data systems, poor cross-sector collaboration, and a lack of documentation on uncertainty and sector-specific risk perceptions. This creates a significant knowledge gap about the extent, causes, and uncertainty of HPAI H5N1 transmission risks in LBMs at the district-level in Indonesia, as well as the practicality of using JRA results to develop effective local risk management and communication strategies.

This study aimed to perform a district-level, multisectoral JRA of HPAI A(H5N1) at the human–animal–environment interface in LBMs of Bogor District and Municipality, Indonesia. It specifically intended to (i) identify and characterize risk pathways for HPAI transmission specific to the local context in LBMs; (ii) estimate the likelihood, potential impact, and uncertainty of human H5N1 infections over the next 12 months using the FAO–WHO–WOAH JRA Operational Tool; and (iii) develop evidence-based, locally relevant risk management and communication strategies to support One Health–oriented policies and practices at the subnational level.

## MATERIALS AND METHODS

### Ethical approval

This study was approved by the Health Research Ethics Committee of the Faculty of Medicine at Universitas Indonesia–Dr. Cipto Mangunkusumo National General Hospital (Approval No. KET.604/UN2.F1/ETIK/PPM/00/ 02/2024). Participants were fully informed about the study’s aims, procedures, and their voluntary participation, with written consent obtained. Only institutional data, with permission from the relevant agencies, were used. No personal identifiers were collected, and all data were stored in aggregated, de-identified formats.

### Study period and location

This study was conducted over a five-month period from May to September 2025 in Bogor, West Java, Indonesia. The study activities were carried out in accordance with the study objectives within this geographic setting.

### Study design and setting

This study conducted a qualitative participatory JRA following the FAO–WOAH–WHO JRA Operational Tool [12]. The workshop took place in Bogor on May 14–15, 2025. The study focused on the Bogor District and Bogor Municipality due to their high poultry densities, active LBMs, and significant intra-district poultry movement. In Bogor Municipality, four markets sell live poultry, with two featuring permanent stalls and two operated by mobile traders who sell birds from crates outside the markets. In Bogor District, eleven markets offer live poultry, with seven featuring permanent stalls; five of these markets have at least five traders and supply various poultry species.

For this JRA, all two municipal markets with permanent poultry stalls and three major district markets were included because they represent the highest-volume LBM nodes. Bogor District spans 2,991.78 km² and is predominantly rural, whereas Bogor Municipality covers 111.37 km² and is predominantly urban. The two jurisdictions host over 6.8 million residents with markedly different population densities. Poultry movement into LBMs averages around 50 birds per trader per day, with 90.84% (25,610,145 birds) sourced from within Bogor District and 9.16% (2,582,440 birds) originating from Lampung, Banten, and other parts of West Java. Poultry movement increases by up to 25% during Ramadan and Eid al-Adha.

Bogor has a well-documented history of AI virus (AIV) transmission. Recent years have seen several subdistricts affected by outbreaks of HPAI in poultry, with the last confirmed human H5N1 case occurring in 2017. This highlights the ongoing zoonotic threat in the region. Given these factors, Bogor is considered a high-risk area that warrants a district-level JRA.

To ensure data validity and assessment reliability, a pre-workshop phase was held before the main JRA workshop. During this period, stakeholders were asked to prepare and bring relevant data from their sectors aligned with predefined risk assessment goals. This strategy aimed to facilitate data-driven discussions and evidence-based decisions during the workshop. Additionally, researchers with prior studies on AIV circulation in LBMs in Bogor District and Municipality were invited, including those who examined the knowledge, attitudes, and practices (KAP) of live poultry traders in the same region. Their input offered valuable context and scientific backing for the multisectoral deliberations.

The workshop was led by experienced staff from the Directorate of Animal Health and the Directorate of Veterinary Public Health within Indonesia’s Ministry of Agriculture. These facilitators were well-versed in conducting JRA activities and the structured FAO–WOAH–WHO tripartite framework, which helped maintain the risk assessment process’s integrity and consistency.

### Participants and sampling strategy

A multisectoral approach was implemented to include representation from the human, animal, and environmental health sectors, as well as market stakeholders. Participants were chosen through purposive sampling, based on their roles as policymakers, technical officers, or individuals with direct access to sector-specific data. In total, 50 participants from eight stakeholder groups and various government, academic, and market institutions took part in the JRA (Table 1).

**Table 1:** Multisectoral stakeholders and institutional representation involved in the joint risk assessment of highly pathogenic avian influenza H5N1 in Bogor District and Municipality, Indonesia.

No.	Sector	Institution Name	Participants (%)
1	Local Government	Regional Agency for Development Planning, Research, and Innovation (RAPDI)	2
		Bogor City Regional House of Representatives	1
2	Human Health	Bogor City Health Office, Bogor, Indonesia	2
		Bogor Regency Health Office, Bogor, Indonesia	2
		Cibinong Regional General Hospital	2
3	Animal Health	Bogor Regency Fisheries and Livestock Office, Bogor, Indonesia	2
		Bogor City Food Security and Agriculture Office, Bogor, Indonesia	2
4	Environmental Sector	Bogor City Environmental Office, Bogor, Indonesia	2
		Bogor Regency Environmental Office, Bogor, Indonesia	2
5	Trade Sector	Bogor City Office of Cooperatives, Small–Medium Enterprises, Industry, and Trade (DinKUKMDagin)	2
		Office of Industry and Trade, Bogor Regency	2
6	Market Sector	Regional Public Market Company “Tohaga” (Bogor Regency)	2
		Regional Public Market Company “Pakuan Jaya” (Bogor City)	2
		Live bird market traders	10
7	Facilitators	Staff of the Directorate General of Livestock and Animal Health Services, Ministry of Agriculture, Republic of Indonesia	2
8	Academics	Virologists	2
		Epidemiologists	2
		Veterinary Public Health Experts	2
		Public health expert	1
		Live bird market research	6
	Total		50

The assessment evaluated 15 LBMs deliberately chosen based on factors, such as market size, poultry turnover, past AI detection, species diversity, and their significance as distribution centers in Bogor District and Municipality. Participants included decision-makers, technical staff with access to official data, and researchers involved in previous studies on AI, surveillance, or KAP assessments in Bogor’s LBMs. This study is among the first to implement a whole-of-society approach to zoonotic risk assessment in Indonesia, involving non-traditional stakeholders, such as market authorities and poultry traders, alongside government and academic institutions.

Each institution was asked to submit sector-specific datasets, such as surveillance reports, market operation records, environmental sampling results, and poultry movement data, in preparation for the JRA. These datasets were first verified internally by the respective agencies using routine supervision. Any cross-sector discrepancies, like mismatched timelines, different case numbers, or inconsistent market figures, were resolved through clarification meetings held before the workshop. Only verified and harmonized datasets were included in the evidence package for the JRA, ensuring consistency, transparency, and comparability across sectors.

### Pre-workshop data collection and preparation

A comprehensive data collection process was carried out across all participating sectors before the workshop to support an evidence-based assessment. Each institution was asked to gather relevant datasets aligned with the JRA objectives. The collected data included: (i) poultry movement records; (ii) animal health surveillance results from both active and passive surveillance via iSIKHNAS; (iii) human surveillance data for influenza-like illness and severe acute respiratory infection; (iv) environmental sampling outcomes; (v) LBM characteristics such as the number of kiosks, slaughtering methods, species mixing, and waste management practices; (vi) adherence to personal protective equipment (PPE) protocols; and (vii) trader KAP data.

Animal health authorities compiled detailed data on poultry movement, including surplus capacity, sources (90.84% locally sourced), day-old chick flow, and inter-district distribution, all of which were validated through internal record checks. Human health data were verified using standard SKDR procedures. Academic researchers who previously studied the same markets validated the behavioral data, PPE compliance, and KAP results. Any discrepancies were addressed through direct meetings between researchers and government officials, where differences were reviewed and harmonized before the JRA workshop.

### JRA process

**Figure 1:**
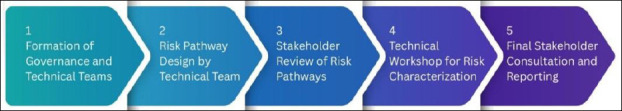
Five-stage process used to implement the joint risk assessment of highly pathogenic avian influenza H5N1 at the human–animal–environment interface in live bird markets of Bogor District and Municipality, Indonesia.

The JRA implementation adhered to a structured five-stage process aligned with the national zoonotic disease risk analysis framework (Figure 1). This study applied the FAO–WHO–WOAH JRA framework by improving pre-workshop data harmonization and using a three-step stakeholder consensus approach, offering a practical model for other high-risk districts.

#### Formation of governance and technical teams

The process began with a virtual coordination meeting attended by representatives from all relevant institutions. The meeting established a steering committee comprising senior officials from the Bogor Municipality Government, the Head of the Bogor City Health Office, the Head of the Livestock and Animal Health Office of Bogor District, and the Director of Perumda Pakuan Jaya. Concurrently, a technical working group was formed, including representatives from key governmental bodies, market administrators, and academic experts.

A stakeholder mapping exercise was conducted to identify and engage additional relevant actors, including poultry traders (high-risk groups), poultry producers’ associations (for data on animal origin and vaccination status), and local nongovernmental organizations (for monitoring wild bird populations). A formal governance structure was established to ensure clear coordination, accountability, and methodological consistency throughout the JRA. This structure comprised three core components: the steering committee, technical working group, and facilitators, each with distinct roles in decision-making, evidence review, and operational oversight (Table 2).

**Table 2:** Governance structure of the JRA, including composition and defined roles of steering committee, technical working group, and facilitators.

Component	Composition	Roles
Steering Committee	Heads of District and Municipal Health Offices; Heads of District Fisheries and Livestock Services; Municipal Food Security and Agriculture Services; District and Municipal Trade Offices; Regional Planning Agencies; Fresh Market Management Authorities; Regional House of Representatives	•Define the JRA scope and objectives • Approve the governance structure • Identifying the JRA Lead • Overseeing the implementation of the assessment • Review and validation of JRA outcomes • Facilitate the adoption of district policy recommendations

Technical Working Group	Mid-level technical officers from the human, animal, environment, trade, and market sectors; academic experts (virology, epidemiology, public health, public health, and LBM)	•Developing and refining risk pathways • Formulation of risk questions • Review and triangulation of evidence • Characterization of likelihood, impact, and uncertainty • Drafting of risk management and communication options • Document all assessment steps

Facilitators	Trained staff from the Directorate General of Livestock and Animal Health Services, Ministry of Agriculture, certified in the FAO–WHO–WOAH JRA Operational Tool	•Lead and coordinate all JRA steps • Adherence to the Operational Tool • Promote balanced multisectoral participation • Facilitates discussions and consensus-building • Documentation of decisions, assumptions, and uncertainties

JRA = Joint risk assessment, LBM = Live bird market, FAO = Food and Agriculture Organization, WHO = World Health Organization, WOAH = World Organization for Animal Health.

Under the leadership of the JRA chairperson, the technical team held a series of virtual meetings to develop preliminary risk pathways, define key risk questions, and identify essential data requirements. Preliminary risk pathways were developed through brainstorming sessions, sticky-note workflows, flipchart diagrams, and FAO pathway templates. The process included hazard identification (HPAI H5N1 virus circulation in poultry), identification of sources, transmission nodes (transportation, holding, slaughtering, and environmental contamination), exposure routes (direct handling, aerosols, fomites, and contaminated waste), and definition of the outcome as human infection at LBMs.

Pathways were validated across all sectors to ensure representativeness and completeness, and assumptions and uncertainties were refined during stakeholder review. The risk question was predefined as the potential for AI transmission in LBMs, based on consistent evidence indicating that LBMs are principal nodes for spillover. Alternative risk questions were not considered, and no inter-sectoral disagreement emerged. A 12-month timeframe was selected to align with Indonesia’s annual planning and budgeting cycle.

#### Stakeholder review of the risk pathways

A multi-stakeholder consultation workshop was held to communicate the JRA objectives and rationale. During the session, the technical team presented preliminary risk pathways, risk questions, and data needs. Stakeholders provided feedback and supplementary information. Evidence integration followed a structured triangulation approach that combined animal surveillance data from iSIKHNAS, human surveillance data from SKDR, environmental sampling results, market operational data (species mixing, slaughter practices, PPE use, and waste management), and expert opinion from researchers and field officers.

#### Technical workshop on risk characterization

During an in-person technical workshop, the team finalized risk pathways and questions, gathered and analyzed relevant data, and created a risk profile. This stage also involved developing risk management and communication strategies, with technical input from the JRA chairperson. Each dataset was validated by its respective sector. Conflicting findings were resolved through consensus discussions, emphasizing data with the most robust verification. Evidence was evaluated qualitatively using the FAO JRA Operational Tool, which provided standardized scoring matrices for assessment. The definitions of qualitative risk levels applied are presented in Table 3, while the impact levels and uncertainty levels are shown in Table 4.

**Table 3:** Standardized qualitative matrix describing likelihood levels used for estimating the probability of human infection with highly pathogenic avian influenza H5N1 in live bird markets.

Likelihood level	Definition
Highly Likely	The event is almost certain to occur within the 12-month assessment period, with strong evidence of active infection sources and repeated exposure.
Likely	The event has a significant chance of occurring; there is some evidence of infection sources and regular exposure.
Unlikely	The event has a low chance of occurring; there is limited evidence of infection sources, though exposure remains possible.
Highly Unlikely	The event is very unlikely; there is no evidence of infection sources or meaningful exposure routes.

The likelihood assessment refers to the probability of at least one human infection occurring within a 12-month assessment period.

**Table 4:** Qualitative criteria used to assess the potential public health, market, and societal impact of human infection with highly pathogenic avian influenza H5N1.

Impact level	Definition
Severe	Multiple human cases (>5), deaths, market closure, large-scale poultry infection, major public panic, and disruption of essential services.
Moderate	Limited number of human cases (3–5), moderate poultry infection, local disruption, and subdistrict-level concern.
Minor	1–2 human cases with no deaths, limited poultry infection, and minimal market impact.
Negligible	No human cases, poultry infection, or operational impact.

Uncertainty levels defined as: • Very low—strong evidence, consistent across sectors • Low–minor data gaps • Moderate: some conflicting or incomplete data. • High–major gaps or unverified assumptions • Very high—insufficient evidence

#### Final consultation and reporting

The final stage included a joint validation meeting with the steering committee and the broader stakeholder group to present finalized risk estimates, interpret findings, and discuss proposed management and commu-nication options. The outcomes informed the finalization of the JRA report. Consensus was achieved through neutral facilitation, ensuring equal participation across sectors. When disagreements arose, evidence was revisited, assumptions clarified, and score adjustments made only when all sectors agreed, ensuring a genuine multisectoral One Health consensus.

### Data sources and tools

All data used in the JRA were collected directly from participating organizations, such as market management authorities, district and municipal animal health offices, public health offices, and regional laboratories. These datasets included iSIKHNAS records, human health surveillance data, environmental sampling results from LBMs compiled by IPB researchers and government agencies, and market operation records. The analysis process, which involved pathway development, evidence synthesis, and risk scoring, was carried out using the FAO–WHO–WOAH JRA Operational Tool along with its standardized templates and matrices. Data organization and compilation were performed in Microsoft Excel, and no other analytical, statistical, or geospatial software tools were employed.

### Limitations of the methodology

This JRA has several limitations. Surveillance of wild birds in the region remains limited, adding uncertainty about viral circulation outside LBMs. The trader KAP survey data may be biased, as self-reported practices might not fully reflect actual behaviors. Moreover, datasets from different agencies vary in completeness and frequency, causing inconsistencies in data quality. While the assessment partly relies on expert opinions, which can be subjective, it is supported by recent field data from researchers working in LBMs, ensuring reasonable accuracy of KAP results and environmental virus monitoring. Despite these issues, triangulating evidence from human, animal, environmental, and market sectors has strengthened the overall validity and robustness of the findings.

## RESULTS

### Risk-framing and scope definition

In the risk-framing stage, the JRA team outlined the assessment’s scope, objectives, and targets to ensure all stakeholders shared a common understanding. The main hazard identified was the HPAI virus type A, subtype H5N1, circulating among domestic poultry. The assessment concentrated on LBMs in Bogor District and Bogor Municipality, where live poultry are sold and slaughtered.

**Figure 2:**
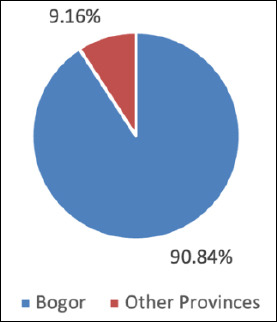
Origin of poultry supplied to live bird markets in Bogor District and Municipality, Indonesia, showing the proportion of locally sourced poultry and poultry originating from other provinces.

A total of 15 LBMs were evaluated, comprising two municipal markets with permanent poultry stalls and three large district markets, which collectively handle over 25 million birds each year. The analysis of poultry movements revealed that 90.8% of birds came from local sources, while 9.2% were imported from Lampung, Banten, and other regions of West Java (Figure 2). Poultry traffic experienced significant seasonal increases of up to 25% during Ramadan and Eid al-Adha.

The study aimed to assess the risk level of HPAI transmission in these markets to support effective risk mitigation strategies. Its main goal was to identify risk management and communication methods to prevent human exposure to HPAI through contact with infected poultry or contaminated market settings. This approach directly shaped the key risk question by examining the likelihood and consequences of human H5N1 infection in high-volume LBMs, taking into account local poultry populations, seasonal trading fluctuations, and poultry movement patterns.

### Identification and mapping of risk pathways

**Figure 3:**
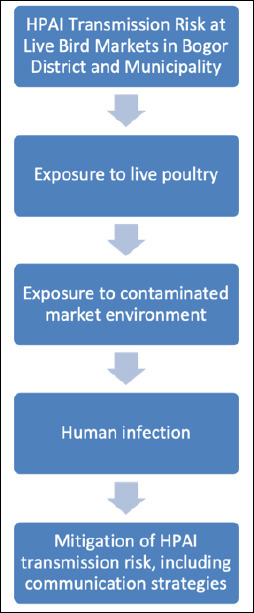
Risk pathway diagram illustrating potential transmission routes of highly pathogenic avian influenza virus type A, subtype H5N1, from poultry to humans within live bird markets in Bogor District and Municipality, Indonesia.

Risk pathways were mapped to describe potential HPAI transmission from poultry to humans through interactions within LBMs. The primary pathway involved the movement of poultry, particularly broilers, layers, and native chickens, into and out of Bogor District and Municipality, with volumes and species composition varying seasonally, especially during religious holidays. Risk pathways illustrating potential HPAI transmission from poultry to humans through interactions within LBMs are presented in Figure 3.

This pathway highlighted key stages, such as poultry transportation, market entry, stall holding, slaughter activities, waste disposal, and direct trader handling. The majority of poultry supplies came from Bogor District, with a smaller share from provinces like Lampung and Banten. While veterinary certification regulated poultry movements, the high daily volume and crowded market conditions posed significant risks for virus transmission.

Secondary or indirect pathways were also identified, such as contamination from transport vehicles, market waste, fomites, and possible contact with wild birds in and around market areas. This JRA highlighted several risk pathways specific to the context, including the heavy dependence on intra-district poultry movement and notable seasonal trading surges, which have not been previously documented in Indonesian HPAI risk assessments.

### Formulation and documentation of the risk question

The next stage involved crafting and officially documenting the primary risk question to ensure all stakeholders shared a consistent analytical framework. The agreed-upon risk question was: “What is the likelihood and impact of at least one person in LBMs in Bogor District and/or Municipality becoming infected with HPAI virus type A, subtype H5N1 through direct or indirect contact with infected poultry or contaminated environments within the next year?”

This question explicitly defined the target population, geographic scope, hazard source, exposure routes, and assessment timeframe, thereby providing a clear basis for risk estimation and management planning. Secondary considerations, including potential economic impacts and the virus’s environmental persistence, were acknowledged for qualitative discussion but were not quantified in this JRA.

The 12-month assessment horizon was chosen to align with Indonesia’s annual planning and budgeting cycle, ensuring that the resulting risk estimates could directly inform near-term policy decisions and mitigation actions. Subpopulations within markets, such as traders, slaughterers, and customers, were considered in risk character-rization, although the primary focus remained on overall human exposure.

### Risk characterization

Risk characterization integrated epidemiological evidence, identified risk pathways, examined trader practices, market conditions, health system capacity, and disease surveillance data. Bogor District had high poultry production, with a population exceeding 28 million birds, and served as a major poultry supplier for surrounding regions. Poultry trading occurred daily under conditions characterized by suboptimal hygiene, limited ventilation, and minimal use of PPE.

Surveillance data from the Bogor District Fisheries and Livestock Office showed that two environmental samples tested positive for influenza A, but no virus isolates were recovered. Additionally, biweekly environmental sampling by researchers from IPB University at five LBMs in Bogor District and Municipality found that the percentage of influenza A–positive samples varied from 78.1% (25 out of 31) to 86.1% (31 out of 36). Importantly, none of these samples tested positive for the H5 subtype.

No human cases of HPAI have been reported in Bogor since 2017, despite the continued high global case fatality rates linked to H5N1. Key exposure risks include poor hygiene among traders, frequent direct contact with poultry, and high human mobility. However, overall uncertainty persists because of incomplete environmental sampling, limited wild bird surveillance, and a lack of data on poultry vaccination compliance.

### Risk estimation

Risk estimation was conducted to assess the likelihood and potential effects of H5N1 infection, aiding evidence-based risk management decision-making. This process enabled a qualitative risk assessment, highlighted uncertainties, and facilitated clear communication of results to stakeholders. It also helped prioritize preventive and response strategies. Details on H5N1 exposure levels in the Bogor area are provided in Table 5, while Table 6 summarizes the potential impact evaluation.

**Table 5:** Criteria for assessing the likelihood of at least one person becoming infected with the H5N1 subtype of highly pathogenic avian influenza virus type A in live bird markets of Bogor within the assessment period (May 2025–April 2026).

Category	Definition	Description
Highly Likely	The event is almost certain to occur within the assessment period (May 2025-April 2026), with strong evidence of active infection sources and repeated exposure.	• Active H5N1 outbreak in poultry at Bogor Market (>30% of poultry infected, ~60 birds/day) • Approximately 200 traders are exposed to slaughtering without PPE daily. • Surveillance confirms poultry cases and high incidence of ILI among traders.

Likely	The event has a significant chance of occurring, with some infection sources and fairly frequent exposure.	• H5N1 was detected in 10%–30% of poultry (~20–60 birds/day). • Traders are frequently exposed through direct (slaughtering) and indirect (dirty cages) contact, with <20% using PPE. • No sporadic poultry deaths were reported in humans.

Unlikely	The event has a low chance of occurring, with limited or no evidence of active infection sources, although potential exposure exists.	• No H5N1 outbreak in Bogor, but endemic in Indonesia. • Traders exposed through risky practices (<20% PPE use, cages cleaned weekly), but no evidence of local poultry infection. • Weak surveillance and no reports of abnormal poultry deaths.

Highly Unlikely	The event is extremely unlikely to occur, with no evidence of infection sources or significant exposure.	• No evidence of H5N1 infection in Bogor or Indonesia • Traders consistently use PPE (>80%), and the market maintains optimal hygiene. • Strong surveillance with negative H5N1 testing in poultry and the environment.

LBMs = Live bird markets, HPAI = Highly pathogenic avian influenza, PPE = Personal protective equipment, ILI = Influenza-like illness.

**Table 6:** Criteria for assessing the potential impact of at least one human infection with highly pathogenic avian influenza virus type A, subtype H5N1, in live bird markets of Bogor.

Impact estimate	Criteria
**Severe**	• >5 human H5N1 cases with >1 death • Overcapacity at the district hospital, depleted oseltamivir stock • >30% of infected poultry (>60 birds/day) • Market closure for >2 weeks • Widespread disruption of food supply • Widespread public panic in Bogor • >50% decline in the number of market visitors

**Moderate**	• 3–5 human H5N1 cases, with ≤1 death • 10%–30% of poultry infected (~20–60 birds/day) • Panic at the sub-district-level • >30% decline in the number of market visitors

**Minor**	• 1–2 human H5N1 cases among traders (no deaths) • <10% of poultry infected (<20 birds/day) • Local culling with no further spread • Disruption <1 week, losses affecting <50 traders • The supply chain recovers quickly • Panic limited to traders • <10% decline in the number of market visitors

**Negligible**	• No human H5N1 cases • No H5N1 infection in poultry • No operational market disruption • No trader losses • No public panic • Public confidence remains stable

Impact assessment incorporates human morbidity and mortality, poultry infection rates, market disruption, supply chain effects, and public perception.

The probability of virus transmission from animals to humans was qualitatively assessed based on expert judgment. To improve transparency, each stakeholder group independently assigned likelihood scores, resulting in the following: Animal Health = Moderate, Public Health = Low, Environmental Sector = Moderate, Market Sector = Moderate, and Trade Sector = Low. A significant methodological aspect of this JRA was the clear documentation of discrepancies in scoring across sectors before reaching a consensus. These variations reflected sector-specific views influenced by data access, practical experience, and operational roles.

To minimize subjectivity, likelihood categories were categorized as high, moderate, low, or negligible, and uncertainty was classified as very high, high, moderate, low, or very low. The data sources used for estimation included poultry movement records, environmental samples from LBMs, surveillance data for animals and humans, and previous KAP studies among market workers.

**Figure 4:**
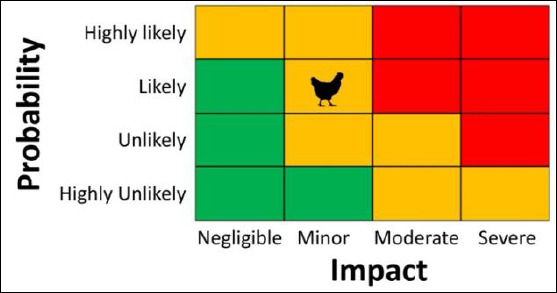
Qualitative risk assessment matrix depicting the likelihood and potential impact of at least one human infection with highly pathogenic avian influenza virus type A, subtype H5N1, in live bird markets of Bogor District and Municipality, Indonesia. Red indicates the need for additional risk mitigation measures and enhanced surveillance; yellow indicates the need to review and adjust existing mitigation measures with targeted surveillance; green indicates that current mitigation measures and routine surveillance can be maintained.

The impact assessment showed a low likelihood of animal-to-human transmission, as there are no confirmed human HPAI cases locally. However, moderate uncertainty persists due to limited surveillance data and reliance on expert judgment. The final risk estimates were incorporated into a qualitative risk matrix, as shown in Figure 4.

Considering the overall risk assessment, the chances of at least one human HPAI infection happening in LBMs in Bogor over the next year are low, with only minor effects on the population. Nevertheless, ongoing vulnerabilities, such as poor hygiene, limited biosecurity, close human–poultry contact, and gaps in surveillance, underscore the need for continued monitoring. These results highlight the importance of enhancing mitigation strategies, broadening surveillance efforts, and improving communication about risks among key stakeholders.

## DISCUSSION

### Novelty and methodological contribution

This study is among the first JRAs of HPAI A(H5N1) conducted at the human–animal–environment interface in Indonesia. It specifically focuses on LBMs in the Bogor District and Municipality. The results show a currently low risk of human infection but also highlight significant structural and behavioral vulnerabilities that could lead to future outbreaks if not addressed. Unlike broader national JRAs that often rely on aggregated surveillance data, this assessment incorporates detailed, market-level epidemiological, behavioral, and environmental evidence, significantly enhancing the precision of risk analysis.

Compared with JRAs conducted in Bangladesh, Vietnam, Egypt, and China, which often rely on sector-specific scoring or surveillance inputs before consolidation, the Bogor JRA featured a more robust, explicit, multisectoral consensus process [14–17]. Risk estimation was carried out through facilitated group discussions and structured consensus among representatives from human health, animal health, and environmental sectors, who collaboratively evaluated evidence, addressed uncertainties, and finalized risk levels [18]. This organized method, supported by existing local governance mechanisms, enabled quick data exchange, balanced deliberation, and shared ownership of outcomes. The study also reveals a new operational alignment between the Tripartite JRA Operational Tool and Indonesia’s national zoonosis coordination framework, offering a practical model for decentralized health systems. An additional finding was the unexpectedly high intra-district poultry circulation during festive seasons, illustrating the dynamic nature of local poultry networks and emphasizing the need for flexible, locally tailored One Health risk assessments.

### Implications for risk mitigation and policy

The findings highlight clear opportunities for feasible risk mitigation. Short-term interventions, including trader education, improvements in environmental sanitation, and adoption of low-cost biosecurity measures, appear achievable with limited resources. In contrast, longer-term interventions, such as market infrastructure upgrades, regulatory reforms, and the establishment of dedicated market health cadres, require sustained political commitment and financial investment. Potential barriers include inconsistent enforcement of regulations, variable trader compliance, limited resources, and behavioral resistance to new practices. These insights provide policymakers with practical guidance on prioritizing interventions based on feasibility and resource availability.

### Relevance of risk-framing and market focus

Risk-framing established a clear, realistic scope by focusing on LBMs as critical nodes in HPAI transmission, consistent with evidence that such markets facilitate viral amplification and environmental persistence [19]. The prioritization of LBMs aligns with recommendations from the WHO, FAO, and WOAH, which identify these markets as key surveillance points for zoonotic influenza [20]. This focus enabled targeted analysis of risk pathways most relevant to human exposure.

### Interpretation of identified risk pathways

Risk pathway mapping showed that while most poultry sold in Bogor comes from local sources and is certified by veterinary authorities, the large daily movement of poultry, especially during festive seasons, significantly raises the risk of introducing infected birds into markets. This aligns with evidence indicating that high poultry turnover and density increase the likelihood of H5N1 circulation in markets [21]. The pathway analysis also pinpointed key control points, such as market entry inspections and environmental hygiene measures, where targeted interventions could be most impactful.

### Risk characterization and uncertainty

Defining a specific risk question offered a focused approach for assessing likelihood and impact. The low probability and minor effects at the population-level reflect the absence of confirmed human HPAI cases in Bogor since 2017 and limited environmental detection of H5 viruses. However, the high global case fatality rate of H5N1 [22] highlights the potentially severe consequences of undetected spillover, underscoring the need for ongoing vigilance despite the currently low risk.

The risk assessment identified significant vulnerabilities, including inadequate hygiene, low PPE compliance, limited environmental disinfection, and insufficient surveillance of poultry and humans. Similar issues have been observed elsewhere and are known to increase virus persistence and human exposure [23]. Evidence shows that AIV contamination in LBMs is closely linked to specific practices, including keeping ducks on-site, failing to isolate sick birds, sourcing poultry via intermediaries, relying solely on water for cleaning, allowing unrestricted dog access, open waste disposal, and nearby wild birds [24]. The moderate uncertainty rating reflects challenges, such as limited surveillance, sporadic testing, and reliance on expert opinion rather than systematic monitoring. By clearly noting sources of uncertainty, such as scarce wild bird data, incomplete poultry vaccination records, and varying hygiene standards, this study offers a transparent approach to communicating uncertainty in JRA. While government virus monitoring remains limited, it is supported by complementary research-based environmental surveillance.

### Implications for One Health–based risk management

The results highlight clear opportunities for improvement through specific risk management and commu-nication measures. A major contribution of this assessment is its focus on feasible, low-cost mitigation strategies tailored for resource-limited environments, providing policymakers with a practical guide. Short- and medium-term actions, such as trader education, enhanced biosecurity, and improved sanitation, are expected to lower risks cost-effectively. For long-term success, strategies such as regulatory reform, improved market infrastructure, and dedicated market health teams are essential for ongoing risk reduction. Evidence shows that simple interventions, such as increasing cleaning frequency, providing access to running water, and avoiding mixing different poultry species in the same cages, can greatly reduce the presence of influenza viruses in market stalls [14]. Other practices, like cleaning poultry transport vehicles, preventing co-sales of waterfowl and chickens, sourcing birds directly from farms, isolating sick birds, and blocking wild bird access, such as house crows, are also linked to lower contamination risks.

## CONCLUSION

This district-level JRA demonstrated that the likelihood of at least one human infection with HPAI A(H5N1) occurring in LBMs of Bogor District and Municipality within a 12-month period is low, with a minor population-level impact and moderate uncertainty. The assessment identified LBMs as persistent but manageable risk nodes, characterized by high poultry turnover, seasonal surges in movement, close human–poultry contact, and suboptimal hygiene and biosecurity practices. Environmental surveillance detected influenza A virus circulation in market settings, although no H5 subtype and no human cases have been reported locally since 2017, indicating limited immediate spillover risk alongside underlying vulnerabilities.

The findings provide actionable, locally relevant evidence to support One Health–oriented decision-making at the subnational level. Immediate, low-cost interventions, such as trader education, routine cleaning and disinfection, improved waste management, and enhanced PPE use, can be implemented with existing resources. Medium-term actions, including enhanced environmental surveillance, improved coordination between human and animal health sectors, and standardized market hygiene practices, are critical to reducing uncertainty and sustaining risk reduction. The results can directly inform district planning, resource allocation, and risk communication strategies tailored to LBMs as priority intervention sites.

Key strengths include a detailed focus at the market-level and the use of a structured, multisectoral JRA framework in practical settings. Combining epidemiological, environmental, behavioral, and operational data from human, animal, environmental, and market sectors improved risk analysis. Clear documentation of sector-specific risk perceptions and efforts to build consensus further increased credibility and policy relevance.

Limitations include sparse wild bird surveillance and incomplete, periodic environmental sampling, contributing to moderate uncertainty. Reliance on expert opinion and self-reported practices may introduce subjectivity or response bias, and variability in data completeness across sectors constrained quantitative interpretation. Multisectoral triangulation mitigated these constraints and supported balanced conclusions.

Future work should prioritize routine and targeted environmental surveillance in LBMs, expanded wild bird monitoring, and improved documentation of poultry vaccination and biosecurity compliance. Periodic repetition of district-level JRAs is recommended to track evolving risks, evaluate intervention effectiveness, and support adaptive management. Assessing behavioral change among traders following interventions will be essential for long-term sustainability.

Overall, although the current risk of HPAI A(H5N1) spillover to humans in Bogor’s LBMs is low, persistent structural and behavioral vulnerabilities warrant proactive mitigation. Strengthening One Health collaboration at the local-level is essential to prevent future outbreaks. This study demonstrates the feasibility and value of district-level JRAs as practical tools for evidence-based zoonotic risk management and offers a replicable model for other endemic, resource-limited settings.

## DATA AVAILABILITY

All the generated data are included in the manuscript.

## AUTHORS’ CONTRIBUTIONS

ES: Conceptualization, project administration, data curation, formal analysis, and writing of the original manuscript. SA: Conceptualization, data curation, and formal analysis, and wrote, reviewed, and edited the manuscript. HP and DI: Project administration, data curation, formal analysis, and writing, review, and editing of the manuscript. SI and GS: Conceptualization and writing, review, and editing of the manuscript. NLPIM, ONP, CB, and YR: Data curation, formal analysis, and reviewed the manuscript. NH: Data curation, formal analysis, and writing of the original draft, review, and editing. All authors have read and approved the final version of the manuscript.
